# Estrogen Receptor β (ESR2) Transcriptome and Chromatin Binding in a Mantle Cell Lymphoma Tumor Model Reveal the Tumor-Suppressing Mechanisms of Estrogens

**DOI:** 10.3390/cancers14133098

**Published:** 2022-06-24

**Authors:** Dan Huang, Zhiqiang Huang, Rajitha Indukuri, Chandrashekar Bangalore Revanna, Mattias Berglund, Jiyu Guan, Konstantin Yakimchuk, Anastasios Damdimopoulos, Cecilia Williams, Sam Okret

**Affiliations:** 1 Department of Biosciences and Nutrition, Karolinska Institutet, SE-141 83 Huddinge, Sweden; dan.huang@ki.se (D.H.); zhiqiang.huang@ki.se (Z.H.); rajitha.indukuri@scilifelab.se (R.I.); chandrashekarbr8@gmail.com (C.B.R.); mattias.berglund@ki.se (M.B.); jiyu.guan@outlook.com (J.G.); konstantin.yakimchuk@ki.se (K.Y.); cecilia.williams@scilifelab.se (C.W.); 2 Department of Protein Science, Science for Life Laboratory, KTH Royal Institute of Technology, SE-171 21 Solna, Sweden; 3 Bioinformatics and Expression Core Facility, Department of Biosciences and Nutrition, Karolinska Institutet, SE-141 83 Huddinge, Sweden; anastasios.damdimopoulos@ki.se

**Keywords:** mantle cell lymphoma, estrogens, estrogen receptor β, ESR2, xenograft, ibrutinib, RNA sequencing, chromatin immunoprecipitation, tumor microenvironment, macrophages

## Abstract

**Simple Summary:**

Mantle cell lymphoma (MCL) is much more common in males than in females. The reason for this is not clear, but research has indicated that the female sex hormones, estrogens, have a protective effect on MCL development. To study this further, mice were transplanted with MCL cells and treated with an estrogen that selectively activates ESR2, the main nuclear estrogen receptor in lymphoma cells. The activation of ESR2 resulted in reduced MCL tumor growth of MCL tumors that were both sensitive and resistant to a newly developed drug (ibrutinib). The mechanism for this effect was investigated by analyzing gene expression and ESR2 binding to target genes. The results show that the affected genes were enriched in several malignancy-related biological processes, including MCL. Furthermore, the results suggested an interplay between the lymphoma cells and the tumor microenvironment in response to ESR2 activation. Altogether, the results clarify the mechanisms of ESR2-mediated MCL growth impairment by estrogens and provide a possible explanation for the sex difference in incidence. Furthermore, targeting ESR2 may be an option when considering the treatment of MCL.

**Abstract:**

Mantle cell lymphoma (MCL) is a non-Hodgkin lymphoma with one of the highest male-to-female incidence ratios. The reason for this is not clear, but epidemiological as well as experimental data have suggested a role for estrogens, particularly acting through estrogen receptor β (ESR2). To study the ESR2 effects on MCL progression, MCL cells sensitive and resistant to the Bruton tyrosine kinase inhibitor ibrutinib were grafted to mice and treated with the ESR2-selective agonist diarylpropionitrile (DPN). The results showed that the DPN treatment of mice grafted with both ibrutinib-sensitive and -resistant MCL tumors resulted in impaired tumor progression. To identify the signaling pathways involved in the impaired tumor progression following ESR2 agonist treatment, the transcriptome and ESR2 binding to target genes were investigated by genome-wide chromatin immunoprecipitation in Granta-519 MCL tumors. DPN-regulated genes were enriched in several biological processes that included cell–cell adhesion, endothelial–mesenchymal transition, nuclear factor-kappaB signaling, vasculogenesis, lymphocyte proliferation, and apoptosis. In addition, downregulation of individual genes, such as SOX11 and MALAT1, that play a role in MCL progression was also observed. Furthermore, the data suggested an interplay between the lymphoma cells and the tumor microenvironment in response to the ESR2 agonist. In conclusion, the results clarify the mechanisms by which estrogens, via ESR2, impair MCL tumor progression and provide a possible explanation for the sex-dependent difference in incidence. Furthermore, targeting ESR2 with a selective agonist may be an additional option when considering the treatment of both ibrutinib-sensitive and -resistant MCL tumors.

## 1. Introduction

Mantle cell lymphoma (MCL) is one of the non-Hodgkin lymphoma (NHL) subtypes, and it constitutes approximately 7% of adult NHL, with an overall poor prognosis [1]. Furthermore, MCLs are heterogeneous regarding their response to targeted therapy using ibrutinib, an inhibitor of the Bruton tyrosine kinase (BTK), a central mediator of B-cell receptor (BCR) signaling. Likewise, for most NHL including MCL, males are more affected than females with male-to-female incidence ratios for MCL varying between 2 and 7:1 in different reports [2,3]. Furthermore, male sex has, in an observational study, been identified as an independent negative prognostic factor [4]. The reason for the sex difference is unclear, but epidemiological data have suggested that estrogens may exert a protective effect on NHL [5,6].

The biological actions of estrogens are largely mediated through nuclear estrogen receptor α (ESR1) and estrogen receptor β (ESR2). Acting as ligand-regulated transcription factors, these receptors, when bound to estrogens, either stimulate or repress target gene transcription. This is performed by binding directly to the DNA at estrogen responsive elements (ERE) by target genes or indirectly by tethering to other transcription factors bound to the DNA [7]. In addition, estrogens may act through G-protein-coupled estrogen receptor 1 (GPER1), a membrane-bound receptor mediating mainly rapid non-genomic responses [8]. Though human lymph nodes express both ESR1 and ESR2, the predominantly expressed nuclear estrogen receptor in normal lymphocytes and lymphomas is ESR2 [9,10]. In addition, GPER1 is expressed in lymphocytes [11] and lymphoma cells [12]. Based on the structural differences in the ligand-binding domains of ESR1 and ESR2, synthetic ligands have been developed, which show receptor selectivity in contrast to 17β-estradiol. One such highly potent selective ESR2 agonist is diarylpropionitrile (DPN), which exhibits a 70-fold binding selectivity for ESR2 over ESR1 and has a 170-fold selectivity of ESR2 transcriptional activation over ESR1 when tested on an ERE-controlled reporter plasmid in transfection experiments [13]. DPN does not activate GPER1 [14].

Estrogen’s effects on the immune system and on lymphocytes are well-recognized [15]. However, the role on lymphomas is less well-established. In order to clarify the molecular mechanisms involved in the ESR2-mediated effects on MCL tumors, the transcriptome in Granta-519 MCL tumors in response to DPN was studied, as well as genome-wide ESR2-binding chromatin sites, using chromatin immunoprecipitation sequencing (ChIP-seq). A deeper understanding of the molecular effects exerted by estrogens on MCL may help to elucidate the mechanisms for the sex-mediated differences in incidence and prognosis. Furthermore, increased knowledge of the genes and signaling pathways affected by estrogens may help to identify new treatment targets.

## 2. Materials and Methods

### 2.1. Cell Lines

The human MCL cell lines Granta-519 (ACC432), Mino (ACC687), JeKo-1 (ACC553), and Maver-1 (ACC717) were obtained from the German Collection of Microorganisms and Cell Cultures GmbH (DSMZ, Leibniz Institute, Braunschweig, Germany). Z-138 (CRL-3001) was obtained from the American Type Culture Collection (ATCC, Manassas, VA, USA). Cells were maintained as previously described [16,17]. The MCL cells were free of mycoplasma infection, and cell identity was verified by STR genotyping at NGI-Uppsala, SciLifeLab, Sweden.

### 2.2. Mice and In Vivo Experiment

Immunocompromised non-obese diabetic severe combined immunodeficiency NOD/SCID IL2γ^null^ (NOD.Cg-*Prkdc^scid^ Il2rg*^tm1Wjl^/SzJ) mice (referred to as NSG mice) were obtained from the Jackson Laboratory (Bar Harbor, ME, USA) and bred at the Animal Facility of Karolinska University Hospital (Huddinge, Sweden). For the xenograft experiment, male mice (8–10 weeks of age) were injected subcutaneously with 15 × 10^6^ MCL cells in 100 µL of sterile PBS in the right flank. When the MCL tumor size reached 100 mm^3^, mice were randomly selected into groups and injected once per day subcutaneously with vehicle, 12.5 µmol/kg body weight of the ESR2-selective agonist DPN (Tocris Bioscience, Bristol, Great Britain, Cat. No. 1494), and/or ibrutinib (Selleck Chemicals, Houston, TX, USA, PCI-32765, Cat. No. S2680, 5 mg/kg body weight), as described earlier [17] and in the figure legend. The tumor size was measured daily and calculated as 0.5 × length (mm) × width^2^ (mm) using a caliper. Mice were sacrificed at the same number of days following the start of treatment except in the case of Granta-519 MCL tumors, where mice were sacrificed when the tumor size of both the DPN- and vehicle-treated mice reached 1300 mm^3^. For a further analysis of the Granta-519 MCL tumors, half of the tumor tissue was fixed in 4% formaldehyde dissolved in PBS for 24 h then transferred to 70% ethanol and stored at 4 °C for TUNEL and Ki67 staining. The remaining half of the tumor tissue was cut into small pieces and kept in RNAlater (Sigma Aldrich, St Louis, MO, USA, Cat. No. R0901) at −20 °C before RNA isolation. All animal experiments were approved by the Swedish Research Animal Ethics Committee (approvals No. 192-14 and No. 14912-2019) and were performed according to the guidelines of the Karolinska Institutet.

### 2.3. Library Preparation, RNA Sequencing, and Data Analysis

RNA sequencing was performed in the Granta-519 tumor samples’ RNA. The Bioinformatics and Expression Analysis (BEA) core facility at Karolinska Institutet prepared the RNA libraries using a TruSeq Stranded mRNA sample prep kit. The libraries were sequenced as single-end 50 bp read lengths on an Illumina HiSeq 2500. The RNA-seq analysis is described in the supplementary methods. The sequence data are uploaded to GEO (GSE190600).

### 2.4. Chromatin Immunoprecipitation (ChIP) and ChIP-Seq Analysis

The ChIP assay was performed as described previously [18] using a highly validated human ESR2 antibody (R & D systems, Minneapolis, MN, USA, Cat. No. PP-PPZ0506-00, 2 µg of antibody per 100 µg of chromatin DNA) on extracts from Granta-519 cells stably over-expressing full-length wild-type ESR2. The Bioinformatic and Expression Analysis core facility at Karolinska Institutet prepared the ChIP DNA libraries using an NEB Next Ultra II DNA Library Prep kit, and quality control was performed using an Agilent TapeStation 2200. The libraries were sequenced on NextSeq 2000 P3 reagents (100 cycles). The ChIP-seq analysis is described in the supplementary methods. The sequencing data are uploaded to GEO (GSE190599).

### 2.5. Statistical Tests

The statistical tests used are described in the Materials and Methods in relation to each method used and complemented with information in the figure legend.

## 3. Results

### 3.1. The ERβ-Selective Agonist DPN Inhibits Growth In Vivo of Both Ibrutinib-Sensitive and -Resistant MCL Grafted to Mice

**Figure 1:**
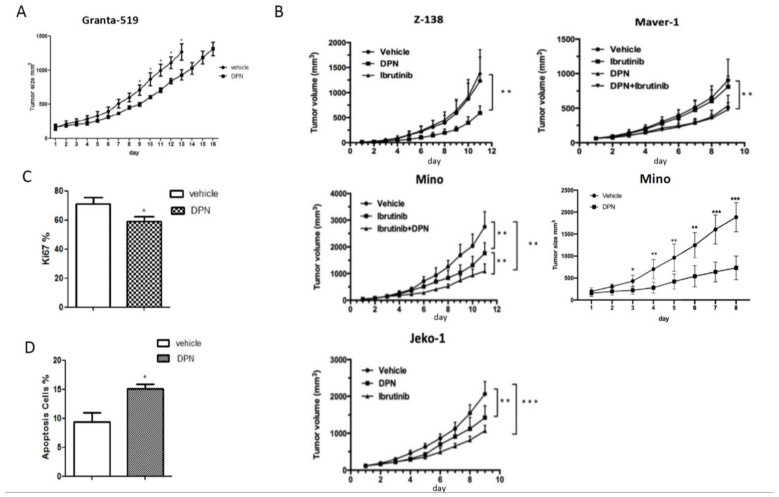
The ESR2 selective-agonist DPN inhibits growth in vivo of both ibrutinib-sensitive and -resistant MCL grafted to mice. (**A**) Male NSG mice were injected subcutaneously with Granta-519 cells and treated subcutaneously daily with vehicle or the ESR2-selective agonist DPN (12.5 mmol/kg body weight). Both groups consisted of 7 mice. Tumor size was measured daily. (**B**) Mino, Maver-1, Z-138, or JeKo-1 cells were engrafted into male NSG mice and treated subcutaneously daily with vehicle, ibrutinib (5 mg/kg body weight), DPN (12.5 mmol/kg body weight), or with both drugs. Tumor size was measured daily. Each group consisted of 6–9 mice, depending on the grafted tumor cell. (**C**) Ki67 immunostaining in sectioned Granta-519 tumors at the size of 1.3 cm^3^. (**D**) TUNEL staining in sectioned Granta-519 tumors at the size of 1.3 cm^3^. The percent of stained cells from 10 randomly chosen fields (at 200× magnification) were counted for each sample. Data are shown as means ± SD. Unpaired two-tailed *t*-test was used for statistical analysis between the two groups, * *p* < 0.05, ** *p* < 0.01, *** *p* < 0.001.

Ibrutinib does not significantly affect the proliferation of ibrutinib-resistant Granta-519 MCL cells [19]. However, the growth curve of Granta-519 MCL grafted to mice showed that tumor progression was impaired in the mice treated with DPN in comparison to when mice were treated with vehicle (Figure 1A). Likewise, DPN impaired the tumor progression of ibrutinib-resistant Z-138 and Maver-1 MCL tumors as well as ibrutinib-sensitive Mino and JeKo-1 MCL tumors (Figure 1B). In ibrutinib-sensitive MCL tumors, DPN and ibrutinib exerted an additive effect. An analysis of the proliferative activity in the Granta-519 tumors by immunostaining for Ki67 demonstrated that proliferation was decreased, whereas a TUNEL assay showed that apoptosis was enhanced (Figure 1C and Figure 1D, respectively).

### 3.2. Transcriptome and Signaling Pathway Analysis of the Granta-519 MCL Tumor Cells Following DPN Treatment

In order to further understand the mechanisms by which DPN impaired MCL tumor growth, alterations of the transcriptome in response to DPN treatment were analyzed by RNA sequencing (RNA-seq). In addition, the signaling pathways involved were studied in Granta-519 MCL tumors. Since the tumor cells were of human origin and the cells constituting the tumor microenvironment (TME) were of murine origin, we could discriminate between alterations in each of the two tumor compartments.

**Figure 2:**
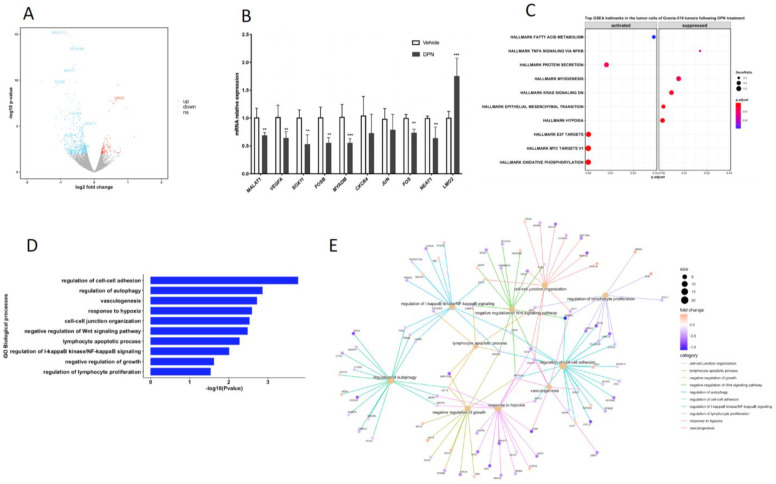
Identification of transcriptome and signaling pathways affected by DPN in the tumor cells of the Granta-519 MCL tumors. (**A**) The volcano plot shows DPN-regulated genes in the tumor cells, as determined by RNA-seq. Genes were considered differentially expressed when *p* < 0.01. Upregulated genes are marked in red, and downregulated genes are marked in blue, while non-significant genes are marked in grey. (**B**) RT-qPCR analysis of 10 selected genes. Results are presented as relative expression (mean ± SD). Unpaired two-tailed *t*-test was used for statistical analysis between the two groups (*n* = 4 to 5, ** *p* < 0.01; *** *p* < 0.001). (**C**) Gene set enrichment analysis (GSEA) using all genes based on fold change. Pathways are sorted by adjust *p* value. (**D**) Gene ontology (GO) biological processes (BP) enrichment analysis of significantly altered genes (*p* < 0.01) in the tumor cells by DPN treatment. (**E**) Gene concept network depicting the linkages of significantly altered genes (*p* < 0.01) and GO terms. Upregulated genes are marked in orange and downregulated genes are marked in blue. The size of a GO BP circle represents the number of genes attributed to it.

The RNA-seq results showed that 538 human genes were significantly affected in the Granta-519 tumors by the DPN treatment (*p*-value < 0.01, Supplementary Table S2). Most (78.6%) of these genes were downregulated (Figure 2A). Based on the RNA-seq differential expression analysis, we selected 10 genes and performed RT-qPCR to confirm the RNA-seq results. The RT-qPCR results showed, in accordance with the results derived from the RNA-seq analysis, that the mRNA expression of LIM domain only 2 (*LMO2*) was significantly increased (*p* < 0.001), while the expression of FOS proto-oncogenes *FOS* and *FOSB*, myeloid-associated differentiation marker (*MYADM*), MCL marker SRY-box transcription factor 11 (*SOX11*), vascular endothelial growth factor A (*VEGFA*), metastasis-associated lung adenocarcinoma transcript 1 (*MALAT1*), and nuclear paraspeckle assembly transcript 1 (*NEAT1*) were decreased (*p* < 0.01) (Figure 2B). The C-X-C motif chemokine receptor 4 (*CXCR4*) and Jun proto-oncogene (*JUN*) showed a tendency for decreased expression, although they did not reach significance in the qPCR analysis.

A gene set enrichment analysis (GSEA), based on ranking all genes according to their fold difference in expression, was performed. Hypoxia, epithelial mesenchymal transition (EMT), and TNFA signaling via NF-κB were among the top-ten most enriched hallmarks, with most genes allocated to these hallmarks being suppressed in the tumor cells following the DPN treatment (Figure 2C and Figure S1A). Among the top-ten hallmarks significantly enriched were also oxidative phosphorylation, MYC targets V1, and E2F targets, with most genes allocated to these hallmarks being activated (Figure 2C and Figure S1B).

To strengthen the analysis of the affected signaling pathways, we also performed an analysis using gene ontology (GO) biological processes (BP) including differentially expressed genes with a *p*-value < 0.01. Enrichment was seen to BP that comprise the regulation of cell–cell adhesion, autophagy, vasculogenesis, the response to hypoxia, cell–cell junction organization, negative regulation of Wnt signaling, the lymphocyte apoptotic process, the regulation of I-κB kinase/NF-κB signaling, and the regulation of lymphocyte proliferation (Figure 2D). The affected genes associated with these BP and their direction of regulation are depicted in the gene concept network (Figure 2E). Most of the genes associated with the “regulation of lymphocyte proliferation” (including *SOX11*) were downregulated, supporting the phenotypic observation in mice with grafted Granta-519 MCL tumors that DPN treatment impaired tumor growth by affecting lymphoma cell proliferation (Figure 1B). The identification of enrichment of the GO BP “lymphocyte apoptotic process” is coherent with the phenotypic observation in the Granta-519 tumors determined by the TUNEL assay (Figure 1C).

### 3.3. Genome-Wide Identification of ESR2 DNA-Binding Regions in the Granta-519 MCL Cells

To identify ESR2 target genes in the Granta-519 MCL cells, we performed a genome-wide characterization of ESR2 chromatin-binding regions by ChIP-seq. Although Granta-519 cells express ESR2 (Supplementary Figure S2A), the expression level was found to be too low to allow for the generation of a clear genome-wide chromatin-binding profile by ChIP-seq when using the most specific and verified ESR2 antibody available, PPZ0506 [9,10], since only a few peaks were identified (Supplementary Figure S3). To overcome this obstacle, we stably overexpressed full-length wild-type ESR2 (530 amino acids, 59 kDa) in the Granta-519 cells using lentivirus-based transduction. The cells, denoted Granta-519-ESR2, expressed approximately 22-fold more ESR2 compared to wild-type Granta-519 cells, which was determined by Western blotting (Supplementary Figure S2A). The transduced ESR2 was shown to be functional, as tested by the ability to effectively induce luciferase expression in response to DPN from a plasmid regulated by estrogen responsive elements (EREs, Supplementary Figure S2B).

**Figure 3:**
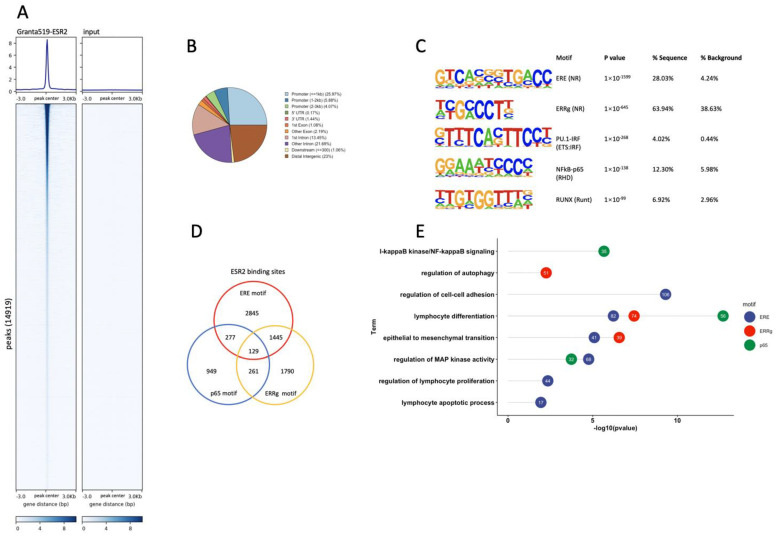
Genome-wide identification of ESR2 binding regions in the Granta-519-ESR2 cells. (**A**) The genome-wide heatmap shows ESR2 peaks’ coverage ±3 kb from the peak center. Each row represents a peak, and the blue color intensity indicates the depth of the signal. (**B**) Genomic distribution of the ESR2 binding regions. (**C**) Top enriched DNA motifs of the ESR2-bound sequences identified using HOMER de novo motif analysis and sorted by *p-*value. (**D**) Venn diagram comparing distribution and co-occurrence of ERE motif, ERRγ motif, and NFκB-p65 motif. (**E**) GO BP enrichment analysis of genes located nearest to ESR2 binding regions with ERE motif, ERRγ motif, and NFκB-p65 motif, respectively. The number in a circle represents the number of genes that belong to this BP.

An analysis of ESR2 binding sites in the Granta-519-ESR2 cells was performed by whole-genome ChIP-seq. A total of 14,919 binding sites were identified in the Granta-519-ESR2 cells following DPN treatment (100 nM for 2 h), which could be attributed to 8689 individual genes. The genome-wide heatmap, Figure 3A, depicts the ESR2 binding sites ±3 kb from the peak center in Granta-519-ESR2 cells and the input sample with a low background signal, demonstrating the specificity of the peaks. Figure 3B illustrates the genomic distribution of the peaks with ESR2 binding, out of which 35.9% were located at promoter regions ≤3 kb from the transcription start site (TSS) and with the majority of these located ≤1 kb from the TSS (Figure 3B).

A search for de novo binding motifs to identify potentially enriched transcription factor binding sites in the Granta-519-ESR2 cells showed that the ERE was the most significantly enriched motif, indicating that the ChIP experiment had good specificity for ESR2 (Figure 3C). In addition, binding motifs for ERRγ, NFκB-p65, PU.1-IRF, and RUNX were also significantly enriched (Figure 3C), indicating that ESR2 might interact with these sites indirectly via tethering to the transcription factors recognizing these motifs directly.

Out of the 14,919 detected binding sites, 31.5% contained the ERE motif, 24.3% contained the ERRγ motif, and 10.8% contained the NFκB-p65 motif (Figure 3D). We next performed a GO BP analysis of the genes that contained these three motifs. Figure 3E shows that genes predicted to be regulated by ESR2 through ERE motifs were enriched for the regulation of cell–cell adhesion, lymphocyte differentiation, EMT, the regulation of MAP kinase activity, the regulation of lymphocyte proliferation, and apoptotic process, whereas genes predicted to be regulated by ESR2 binding to NFκB-p65 were enriched for lymphocyte differentiation, I-κB kinase/NF-κB signaling, and the regulation of MAP kinase activity. Genes predicted to be regulated by ESR2 binding to ERRγ were enriched for lymphocyte differentiation, EMT, and the regulation of autophagy (Figure 3E).

### 3.4. ESR2 Binding Occurs to the DPN-Regulated Genes in the Tumor Cells of the Granta-519 Tumors

**Figure 4:**
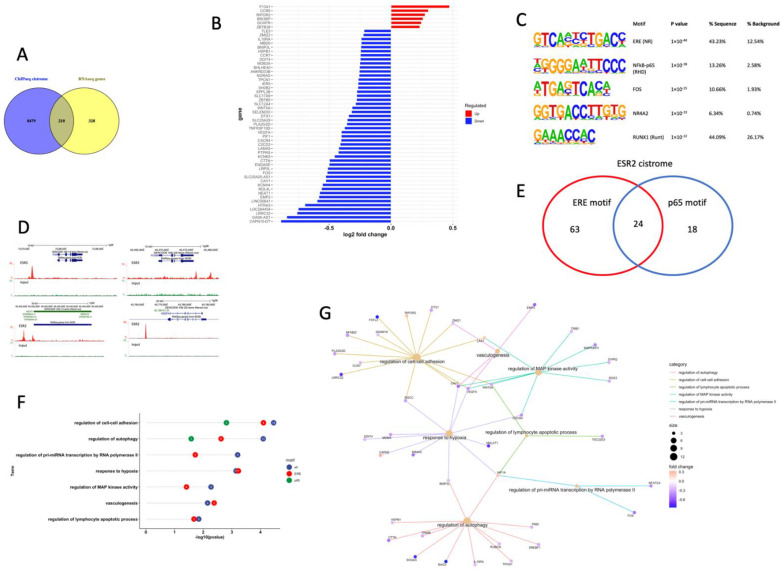
ESR2 binding occurs to the DPN-regulated genes in the Granta-519 tumor cells. (**A**) Venn diagram of ESR2 cistrome and transcriptome data identified 210 differentially regulated genes that showed direct ESR2 binding. (**B**) Bar graph showing the log2-fold change of genes from tumor cells after DPN treatment with peak scores >30 in the ChIP-seq analysis. Upregulated genes are marked in red, and downregulated genes are marked in blue. (**C**) The top enriched DNA motifs in the 411 ESR2-bound sequences present in the 210 genes were identified using HOMER de novo motif analysis and sorted by *p*-value. (**D**) ESR2 ChIP-seq enrichment signals and localization for *FOS*, *FOSB*, *NEAT1,* and *VEGFA* binding sites visualized by UCSC genome browser. (**E**) Venn diagram of ESR2 cistrome with ERE motif and NFκB-p65 motif. (**F**) GO BP enrichment analysis based on the 210 genes that are differentially regulated and have ESR2 binding regions with ERE motif and/or p65 motif, respectively. The number in a circle represents the number of genes that belong to this BP. (**G**) Gene concept network depicting the linkages of significant genes and GO terms. Upregulated genes are marked in orange, and downregulated genes are marked in blue. The size of a GO BP circle represents the number of genes attributed to it.

In order to investigate which genes in the Granta-519 tumors were directly affected by ESR2, the ChIP-seq data derived from the Granta-519-ESR2 cells were combined with the expression results obtained from the RNA-seq analysis of human genes in the Granta-519 tumors. Of the significantly regulated human genes derived from the RNA-seq analysis, 210 genes (39%) were found to harbor ESR2 binding sites (Figure 4A and Supplementary Table S3). Among them were *VEGFA*, *FOS*, *FOSB*, *CXCR4*, *MALAT1,* and *NEAT1*. Figure 4B shows the differential expression of genes with the highest ESR2 binding peak scores (>30), which included the *VEGFA*, *NEAT1*, *FOS,* and *CXCR4* genes. Interestingly, no direct binding of ESR2 to the *SOX11*, *JUN*, and *MYADM* genes was detected, suggesting that these genes are regulated by DPN through an indirect mechanism and not by ESR2 binding.

A de novo motif analysis of the 411 ESR2 binding sites present in the 210 genes showed that binding motifs for the transcription factors ESR (ERE), NFκB-p65, FOS, NR4A2 (NURR1), and RUNX1 were the most enriched (Figure 4C). Figure 4D depicts the position of the ESR2 binding in relation to the *FOS*, *FOSB*, *NEAT1*, and *VEGFA* genes. Among the 210 significantly regulated genes with ESR2 binding sites, 87 genes (41.4%) had the ERE motif and 42 genes (20%) had the NFκB-p65 binding motif, while 24 genes (11.4%) contained both the ERE and NFκB-p65 motifs (Figure 4E and Supplementary Table S4).

A GO BP analysis using the 210 transcriptionally regulated genes with ESR2 binding showed that among the significantly enriched pathways were cell–cell adhesion, autophagy, the response to hypoxia, the regulation of MAP kinase activity, vasculogenesis, and the regulation of the lymphocyte apoptosis process (Figure 4F). This figure also shows that the ERE motif was present in the genes contributing to the enrichment of all these pathways, while the NFκB-p65 motif was found to be restricted to the genes contributing to cell–cell adhesion and autophagy. The affected genes associated to these BPs and their direction of regulation are depicted in the gene concept network (Figure 4G).

### 3.5. ESR2 Binding to the Regulated Genes Is Enhanced following DPN Treatment

**Figure 5:**
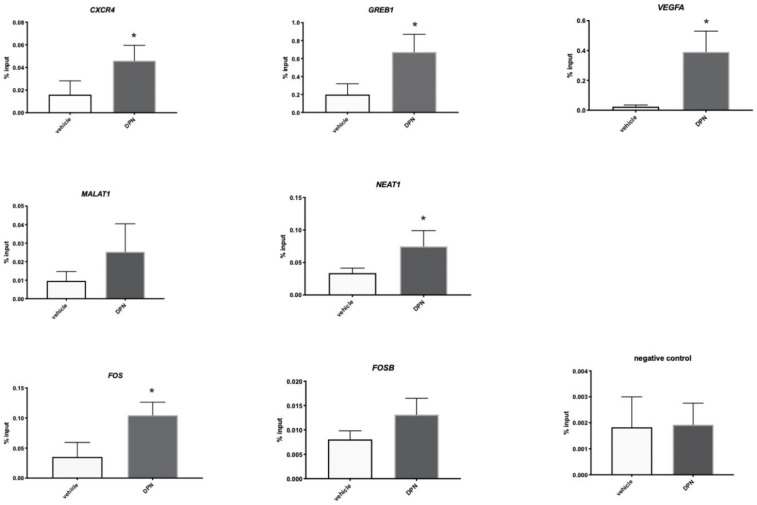
ESR2 binding to the regulated genes is enhanced following DPN treatment. ChIP-qPCR showing ESR2 binding to *CXCR4* (Chr2, position 136117143–136117371), *GREB1* (Chr2, position 11541917–11542145), *VEGFA* (Chr6, position 43760763–43760991), NEAT1 (Chr11, position 65418735–65418963), *MALAT1* (chr11, position 65497575–65497803), *FOS* (Chr14, position 75275317–75275545), and *FOSB* (Chr19, position 45475539–45475767) (Genome version: UCSC human reference genome GRCh38) following a 2 h treatment of the Granta-519-ESR2 cells with 100 nM DPN or vehicle. Data are represented as means ± SD. An unpaired two-tailed *t*-test was used for statistical analysis between the two groups generated from three separate experiments (* *p* < 0.05).

In order to validate if the ESR2 binding of the genes identified in the ChIP-seq increased in response to DPN treatment, Granta-519-ESR2 cells were treated with 100 nM DPN or vehicle for 2 h, after which a selected number of genes, *FOS*, *FOSB*, *VEGFA*, *CXCR4*, *MALAT1,* and *NEAT1*, were analyzed by ChIP-qPCR. *GREB1* was used as a positive control since it was previously shown by ChIP to bind ESR2 when analyzed using the PPZ0506 antibody [18]. When comparing DPN- to vehicle-treated Granta-519-ESR2 cells, the enrichment of ESR2 binding to *CXCR4*, *GREB1*, *VEGFA*, *NEAT1*, and *FOS* was detected following the DPN treatment, demonstrating that the binding of ESR2 to these genes was increased upon ligand binding. ESR2 binding to *MALAT1* and *FOSB* showed a tendency to increase in response to DPN administration but did not reach statistical significance (Figure 5).

### 3.6. DPN Treatment of Granta-519 Cells in Culture Does Not affect Proliferation or Apoptosis

The Granta-519 tumor grafting experiment demonstrated that tumor cell proliferation decreased and apoptosis increased in response to DPN treatment (Figure 1C,D above). In order to investigate if these responses were a direct effect on the tumor cells or required contributions by other cellular components, we treated carboxyfluorescein succinimidyl ester (CFSE)-labeled wild-type or Granta-519-ESR2 cells in culture with 100 nM DPN or vehicle for 5, 7, or 10 days. The results showed that DPN treatment had no direct effect on Granta-519 cell proliferation (Supplementary Figure S4A,B). Furthermore, the apoptosis of Granta-519 cells and Granta-519-ESR2 cells was not significantly affected by 24 h of DPN treatment (Supplementary Figure S4C,D). This is surprising, considering that the cells express ESR2 mRNA [16] and ESR2 protein (Supplementary Figure S2A). To further investigate this, we analyzed the expression of a selected number of genes in the wild-type Granta-519 cells in culture following DPN treatment. Interestingly, some of the genes, but not all, found to be regulated in vivo were also significantly regulated in Granta-519 cells in vitro. This included the ESR2-binding genes *CXCR4*, *FOSB*, and *VEGFA* as well as the non-ESR2-binding genes *MYADM* and *LMO2.* In contrast, the regulation of *FOS*, *MALAT1,* and *SOX11* was not significantly regulated in culture (Supplementary Figure S5). The regulated expression of these latter genes might therefore require the interaction of the Granta-519 lymphoma cells with the TME.

### 3.7. Transcriptome and Signaling Pathway Analysis in the TME of the Granta-519 Tumors Following DPN Treatment

**Figure 6:**
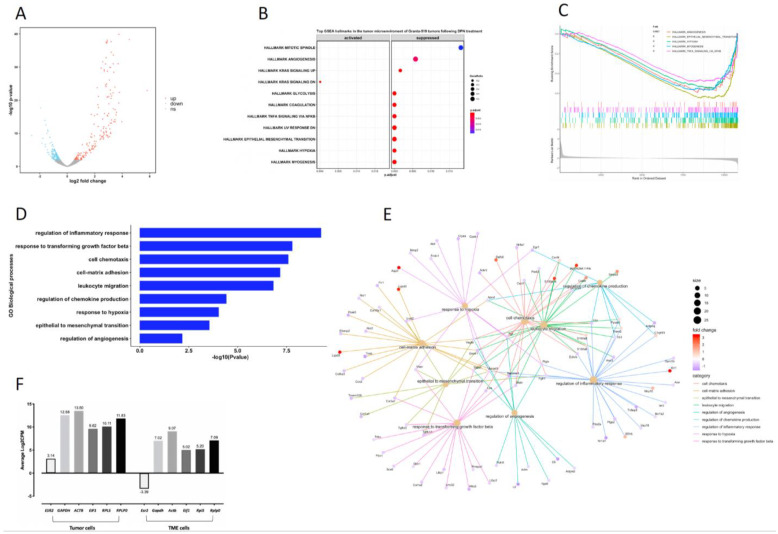
Identification of transcriptome and signaling pathways affected by DPN in the TME of the Granta-519 MCL tumors. (**A**) The volcano plot shows DPN-regulated genes in the TME, as determined by RNA-seq. Genes were considered differentially expressed when *p* < 0.01. Upregulated genes are marked in red, and downregulated genes are marked in blue. Non-significant genes are marked in grey. (**B**) GSEA analysis showing the top pathways affected when using all genes based on log2-fold change. Pathways are sorted by adjusted *p* value. (**C**) Enrichment plot for the top pathways. The data are presented with enrichment scores and adjusted *p*-values. (**D**) GO BP enrichment analysis of significantly altered genes (*p* < 0.01) in the TME by DPN treatment. (**E**) Gene concept network depicting the linkages of significantly altered genes and GO terms. Upregulated genes are marked in orange, and downregulated genes are marked in blue. The size of a GO BP circle represents the number of genes that belong to it. (**F**) The relative expression of *ESR2* and reference genes (*GAPDH*, *ACTB*, *EIF1*, *RPL5,* and *RPLP0*) in tumor cells and their murine homologues in the TME of the Granta-519 tumors based on the RNA-seq results (*n* = 7).

Considering that the response of the tumor cells in the Granta-519 tumors to DPN may rely on the TME, transcriptional alterations in the TME (murine genes) were analyzed. The results showed that a total of 430 genes were significantly regulated (*p* < 0.01), with 57.7% being downregulated after DPN treatment (Figure 6A and Supplementary Table S5). A GSEA of the murine genes showed that among the top 11 significantly affected hallmarks were hypoxia, EMT, TNFA signaling via NF-κB, and angiogenesis, with genes attributed to these hallmarks mainly being suppressed by the DPN treatment (Figure 6B,C). A GO BP analysis based on significantly regulated genes in the TME showed that the significantly affected biological processes were the regulation of the inflammatory response, the response to transforming growth factor beta (TGFβ), cell chemotaxis, cell–matrix adhesion, leukocyte migration, the regulation of chemokine production, the response to hypoxia, EMT, and the regulation of angiogenesis (Figure 6D). The association of significantly regulated genes and their direction of regulation for these BP are depicted in the gene concept network (Figure 6E). Notably, the results showed that most of the genes belonging to the regulation of the inflammatory response, the response to TGFβ, cell–matrix adhesion, the response to hypoxia, EMT, and angiogenesis were downregulated (Figure 6E). These results demonstrate that in addition to the Granta-519 tumor cells, cells of the TME are largely affected by the DPN treatment. It is notable that the *Esr2* expression in the TME is low in comparison to the *ESR2* expression in the tumor cells (Figure 6F).

### 3.8. Changes in Immune Cell Composition of the TME following DPN Treatment

**Figure 7:**
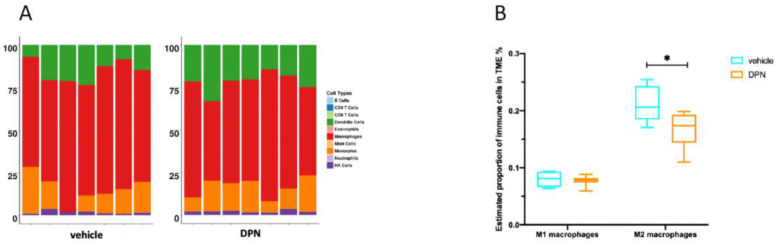
The composition of immune cells in the TME of the Granta-519 MCL tumors. (**A**) Bar plot showing the estimated composition of 10 different immune cells in the TME, as measured by Seq-ImmuCC in individual Granta-519 tumors treated with DPN and vehicle. (**B**) Box and whisker plot showing the estimated composition of M1 and M2 macrophages in Granta-519 tumors treated with DPN and vehicle, respectively, as determined by CIBERSORTx. An unpaired two-tailed *t*-test was used for statistical analysis between the two treatments (*n* = 6–7 per group, * *p* < 0.05).

We next analyzed the changes in the immune cell composition of the TME following the DPN treatment. Using Seq-ImmuCC [20], we observed that the macrophage population was the most abundant immune cell type of the Granta-519 tumor TME in the NSG mice (Figure 7A, note: NSG mice lack lymphocytes). In order to differentiate between M1 and M2 macrophages, we also used CIBERSORTx, which provides information on macrophage subtypes [21]. The results indicated that the number of M2 macrophages was decreased following the DPN treatment, while the number of M1 macrophages was not affected (Figure 7B).

## 4. Discussion

We show that the reduced tumor size seen by DPN treatment following the grafting of Granta-519 MCL cells to mice is due to impaired tumor cell proliferation and increased apoptosis, an effect not due to different tumor sizes. By analyzing the transcriptomes in the tumor cells and the TME as well as genome-wide ESR2 binding, we describe the molecular mechanisms responsible for the impaired tumor progression seen in response to the DPN treatment. The significantly enriched signaling pathways in the lymphoma cells of the Granta-519 MCL tumors in response to the DPN treatment included the regulation of I-κΒ kinase/NF-κB signaling, EMT, cell adhesion, vasculogenesis, the lymphocyte apoptotic process, and the regulation of lymphocyte proliferation. The identification of the biological processes of the “lymphocyte apoptotic process” and the “regulation of lymphocyte proliferation” are consistent with the increase in apoptosis and the decrease in proliferation (including downregulation of *SOX11* expression) observed in the Granta-519 tumors following the DPN treatment. It is notable that a high expression of Ki67 is a strong indicator of poor prognosis in MCL [22].

NF-κB is a transcription factor complex that regulates the growth and survival of B-cells, and MCL has been shown to depend on NF-κB signaling for growth and proliferation [23]. NF-κB has anti-apoptotic effects protecting the tumor cells from undergoing cell death [24]. Furthermore, Saba et al. demonstrated that the constitutive activation of canonical NF-κB signaling via BCR is a key feature found in most MCLs [25]. Therefore, many new drugs for targeting the canonical NF-κB signaling have been introduced for the treatment of MCL, with a good response, at least initially [23]. Thus, a reduction in the canonical NF-κB signaling pathway following DPN treatment can explain the reduced tumor cell proliferation and increased apoptosis seen in MCL tumors. Notably, the ibrutinib-resistant Granta-519, Z-138, and Maver-1 cells [19] responded to the treatment with an ESR2-selective agonist, similar to the ibrutinib-sensitive Mino and JeKo-1 MCL tumors, as demonstrated in this report. The cause of the ibrutinib resistance in MCL cells has been shown to be due to constitutive activation of the non-canonical NF-κB signaling pathway, which is not targeted by BTK or PKC inhibitors, in contrast to the canonical NF-κB signaling, which is sensitive to BTK or PKC inhibitors [19]. Therefore, our results suggest that ESR2 agonists may also be useful for inhibiting ibrutinib-resistant MCLs that rely on non-canonical NF-κB signaling.

The biological process of altered cell–cell adhesion in response to DPN treatment is interesting, as it has been shown that adhesion receptors in lymphoid malignancies contribute to lymphoma aggressiveness by their capability to transduce signals into the cells promoting tumor cell growth and survival as well as enhancing lymphoma dissemination [26]. An altered cell–cell adhesion with regulated genes associated to this biological process mainly being suppressed is consistent with a previous study from us where we showed that DPN treatment of mice that had been subcutaneously grafted with disseminating Burkitt’s Raji lymphoma cells resulted in less liver metastasis [16]. Furthermore, the treatment of Mino and Rec-1 MCL cells grown in the presence of stromal cells with antibodies against the adhesion receptor VLA-4 could overcome the stromal-cell-mediated ibrutinib resistance of the MCL cells, emphasizing a role for cell–cell adhesion in MCL survival [17]. It was also recently shown that the inhibition of BCR signaling disrupts adhesion in MCL, connecting downstream BCR signaling, including NF-κB signaling, to the regulation of cell–cell adhesion [27]. In addition, the GO BP analysis identified vasculogenesis as being significantly altered, which may be explained by a decreased expression of *VEGFA*. EMT was also affected by DPN, with genes associated with this pathway mainly being suppressed. Growing evidence suggests that EMT promotes leukemia and lymphoma progression as well as therapy resistance [28,29]. Moreover, the EMT process is also closely connected to the TME [30,31,32,33].

In addition to the enriched signaling pathways altered by the DPN treatment, individual genes that were significantly altered and were described to be highly relevant in MCL pathogenesis were identified. This included decreased *SOX11* expression. The transcription factor SOX11 has been shown to be a key oncogenic factor in MCL by promoting tumor growth in vivo by regulating B-cell differentiation pathways and controlling the cell cycle and apoptosis [34,35]. Furthermore, *SOX11* overexpression associates with increased signaling via the BCR and can be reversed by BTK inhibition. The impaired tumor progression seen in the Granta-519 MCL tumors following DPN treatment may therefore involve a DPN-mediated suppression of *SOX11* expression. *SOX11* also regulates *CXCR4* expression, with *CXCR4* being important for the tumor–TME interaction [34]. MCL is known to express high levels of the *CXCR4* chemokine receptor, which contributes, similar to VLA-4, to adhesive interactions between MCL cells and stromal cells of the TME, conferring cell survival and drug resistance [36]. Notably, *CXCR4* expression in tumor cells and vasculogenesis in the TME were decreased following DPN treatment, further featuring an MCL tumor cell–TME connection.

The expression of some additional genes described to be relevant for MCL pathogenesis was also found to be regulated by the DPN treatment. This included *MALAT1* and *NEAT1*. Both *MALAT1* and *NEAT1* belong to the group of long non-coding RNA (lncRNA). Wang et al. showed that MALAT1 expression is elevated in human MCL tumors and cell lines compared to normal controls, and the elevated levels of MALAT1 correlated with a higher MCL international prognostic index and reduced overall survival [37]. Furthermore, MCL with a knockdown of MALAT1 expression showed impaired cell proliferation and facilitated apoptosis [37]. NEAT1 may exert a similar effect because a knockdown of NEAT1 inhibits cell proliferation and promotes apoptosis in DLBCL [38]. In colon cancer, NEAT1 activates Wnt/β-catenin signaling, promoting cancer progression [39]. The expression of both *MALAT1* and *NEAT1* were downregulated by DPN in the Granta-519 MCL tumors, according to the RNA-seq analysis.

In order to identify direct ESR2 binding targets, a genome-wide ChIP-seq was performed. The most significant ESR2 binding motif found in the genes transcriptionally regulated by DPN was ERE. Other transfection factor binding motifs found in the ESR2 binding sites were NF-κB, FOS, NR4A2, and RUNX1. This is in line with the finding that most of the above transcription factors binding to the non-ERE-containing motifs have been shown to crosstalk with ESR1 and ESR2. For example, a crosstalk between ESR with NF-κB proteins is well-established and has been suggested to explain the immunosuppressive and anti-inflammatory effects of estrogens [40,41]. Similarly, crosstalk between ESR1 and ESR2 and the AP-1 (FOS/JUN) complex is recognized [18,42,43] as well as with the RUNX transcription factors [44]. A crosstalk between ESR and NR4A2 is less well-established.

One biological process identified as significantly enriched when performing the GO BP based on the significantly regulated genes with ESR2 binding sites was the “regulation of MAPK activity”. MAPK activation is an important downstream signaling pathway of the BCR, and the inhibition of BCR will thus reduce MAPK signaling, including the activity of the downstream MAPK3/1 (also called ERK1/2). ERK1/2 are involved in the transcription of regulatory genes such as *FOS* and *JUN* that are important for cell survival in MCL [45]. This is in line with the *FOS* and *JUN* genes being downregulated by DPN in the Granta-519 tumors, as determined by the RNA-seq.

Interestingly, the pro-apoptotic and anti-proliferative effects seen in Granta-519 cells in vivo were not seen in Granta-519 cells in culture, indicating that the interactions between lymphoma cells and other cellular compartments, such as the TME, are important for these responses. Two of the most significant pathways in the TME that were affected by the DPN treatment were the “regulation of inflammatory response” and the “response to TGFβ”. Most of the genes associated to the “regulation of inflammatory response” were downregulated by the DPN treatment, suggesting a reduced activity of this BP. The BP “response to TGFβ” was also mainly associated with a reduced expression of genes, implicating impaired TGFβ signaling. This was substantiated by a reduced expression of *Smad3*, a central intracellular signaling molecule downstream of the TGFβ receptor [46]. Importantly, TGFβ signaling is an important player in the regulation of the TME and could be ascribed to several of the regulated signaling pathways found to be affected in the TME of the Granta-519 tumors. Dysregulated TGFβ signaling has also been described in MCL [47]. TGFβ and TGFβ receptor signaling promote tumor growth and metastasis as tumors progress by stimulating EMT, inducing myofibroblast differentiation, altering the differentiation and proliferation of immune cells, modulating the matrix composition, and reprograming cell metabolism [46,48]. The reduced TGFβ signaling in the TME may therefore also contribute to the suppression of EMT, angiogenesis, myogenesis, and some of the other enriched hallmarks in the TME identified by the GSEA. It is notable that the reduced tumor progression brought about by DPN treatment does not involve host lymphocytes, as the immunocompromised NSG mice used for the experiments lack B, T and NK cells. However, following the DPN treatment, we observed a reduction in M2 macrophages in the TME, while the M1 macrophages were not affected. Since M2 macrophages promote tumor cell proliferation and invasion [49], this reduction in tumor-associated M2 macrophages may contribute to the reduction in Granta-519 tumor progression following DPN treatment. Furthermore, tumor-associated macrophages can express a variety of cytokines, including TGFβ1, that stimulate tumor cell proliferation and survival. Thus, a reduced abundance of tumor-associated M2 macrophages following DPN treatment relates to the “negative regulation of inflammatory response” and the “response to TGFβ” in the TME of the Granta-159 tumors, as determined by the GO BP analysis. These above results imply that the TME is an important contributor in eliciting the effect of estrogens via ESR2 on MCL. This is likely an indirect effect since *Esr2* expression in the TME is very low.

## 5. Conclusions

Our results clearly demonstrate that MCL is under estrogen control mediated by ESR2, as shown in the grafting experiments of both ibrutinib-resistant and -sensitive MCL cells. The ESR2-mediated responses, as studied in Granta-519 MCL tumors, involve the alteration of several signaling pathways in the tumor cells as well in the TME that help to clarify the mechanism for the impaired tumor progression seen by estrogens. Furthermore, the results may also help to explain the lower incidence of MCL in females compared to males, supporting a protective role. In addition, targeting ESR2 with a selective agonist not interacting with ESR1 (ESR1 being responsible for many of the unwanted side effects, e.g., the growth stimulatory effects on the breast and uterus) may be an additional approach when considering the treatment of MCL, especially ibrutinib-resistant MCL. The results may also lead to a more personalized treatment that takes sex and endocrine status into consideration.

## Data Availability

The transcriptome and ChIP-seq datasets generated in the current project are available in the NCBI Gene Expression Omnibus (GEO) repository (https://www.ncbi.nlm.nih.gov/geo/) with accession No. GSE190600 and GSE190599 (accessible on 30 June 2022), respectively. Additional information is also available as supplementary tables, figures, and methods.

## Supplementary Materials

The following supporting information can be downloaded at https://www.mdpi.com/article/10.3390/cancers14133098/s1, Figure S1: Enrichment plots for the top downregulated (S1A) and upregulated (S1B) pathways in the Granta-519 MCL tumor cells following DPN treatment; Figure S2: (A) Western blot analysis showing ESR2 expression in wild-type Granta-519, Granta-519-ESR2, and Granta-519-mock cells, (B) The transduced ESR2 in Granta-519-ESR2 cells is functional; Figure S3: Genome-wide identification of ESR2 binding regions in Granta-519-mock cells not overexpressing ESR2; Figure S4: The effect of DPN treatment on proliferation and apoptosis of the Granta-519-mock and Granta-519-ESR2 cells in culture; Figure S5: DPN regulates expression of a number of genes in Granta-519 cells in culture; Table S1: Primers used for RT-qPCR and ChIP-qPCR (docx); Table S2: List of human genes significantly affected in the Granta-519 MCL tumors by the DPN treatment (xls); Table S3: List of significantly regulated human genes derived from the RNA-seq which harbor ESR2 binding sites (xls); Table S4: ERE and p65 motifs present in the 210 significantly regulated genes that demonstrate ESR2 binding (xls); Table S5: Genes significantly affected in the tumor microenvironment of the Granta-519 MCL tumors by the DPN treatment (xlsx); Supplementary methods: Supplementary methods that were not described in the research article (docx).
