# Supplementary material for: Estrogen Receptor β (ESR2) Transcriptome and Chromatin Binding in a Mantle Cell Lymphoma Tumor Model Reveal the Tumor-Suppressing Mechanisms of Estrogens
Source: Cancers (Basel). 2022 Jun 24;14(13):3098. doi: 10.3390/cancers14133098 (PMC9264873; doi:10.3390/cancers14133098)
Supplement: Supplementary file 1 [file cancers-14-03098-s001.zip › Supplementary figures S1-S5.pdf]

Suppl. Figure S1

A B

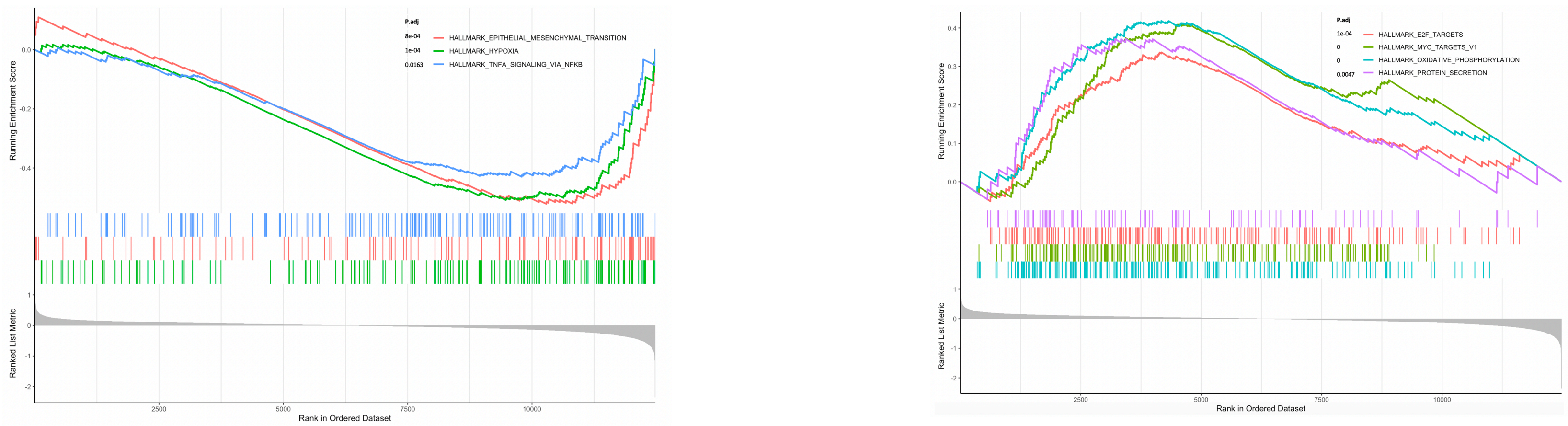

Supplementary figure S1: Enrichment plots for the top down-regulated (S1A) and up-regulated (S1B) pathways in the Granta-519 MCL tumor cells following DPN treatment. Data are presented with enrichment scores and adjust *p* values.

Suppl. Figure S2

A

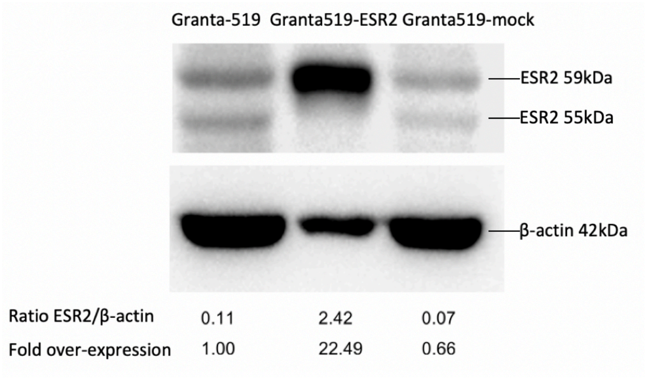

B

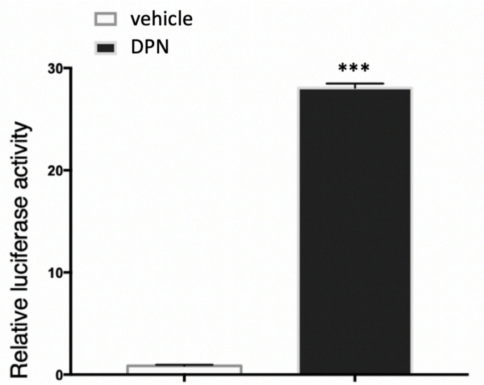

Supplementary figure S2A: Western blot analysis showing ESR2 expression in wild-type Granta-519, Granta519-ESR2 and Granta519-mock cells. The 59kDa band represents the full length ESR2. The lower band is likely due to expression of splice variants of ESR2 with a molecular weight 55kDa. The figure shows a representative blot out of 3 independent experiments.

Supplementary figure S2B: The transduced ESR2 in Granta519-ESR2 cells is functional. Relative luciferase activity of a 3 × ERE-luciferase (firefly) reporter plasmid and pRL-TK Renilla luciferase plasmid (internal control for normalizing transfection efficiency) which were electroporated into Granta519- ESR2 cells prior to treatment with 100 nM DPN or vehicle for 24h. The firefly and renilla luciferase activities were measured by luminometer. Unpaired two-tailed t-test was used for statistical analysis between the two treatments (n=4 per group, ***p<0.001.)

Suppl. Figure S3

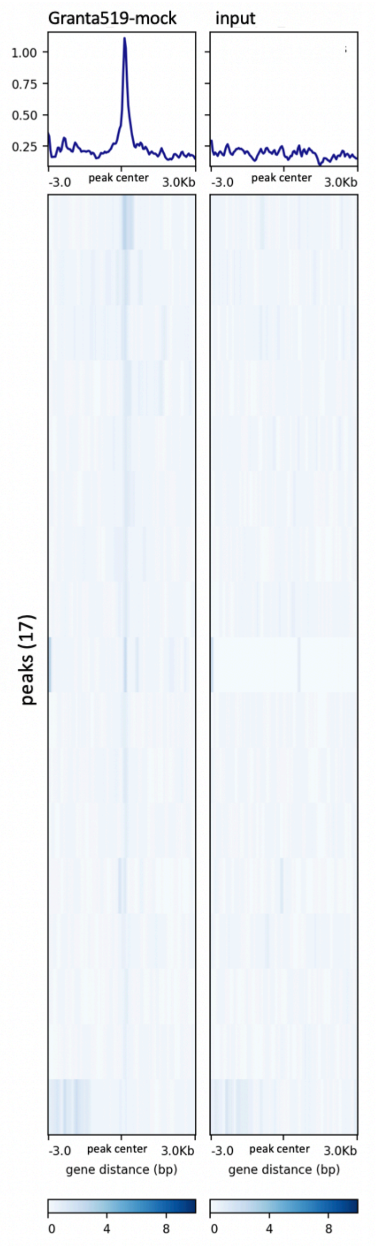

Supplementary figure S3: Genome wide identification of ESR2 binding regions in Granta519-mock cells not overexpressing ESR2. The genome-wide heatmap shows few ESR2 binding sites in the region ±3kb around the peak center. Each row represents a peak and the blue color intensity indicates the depth of the signal.

Suppl. Figure S4

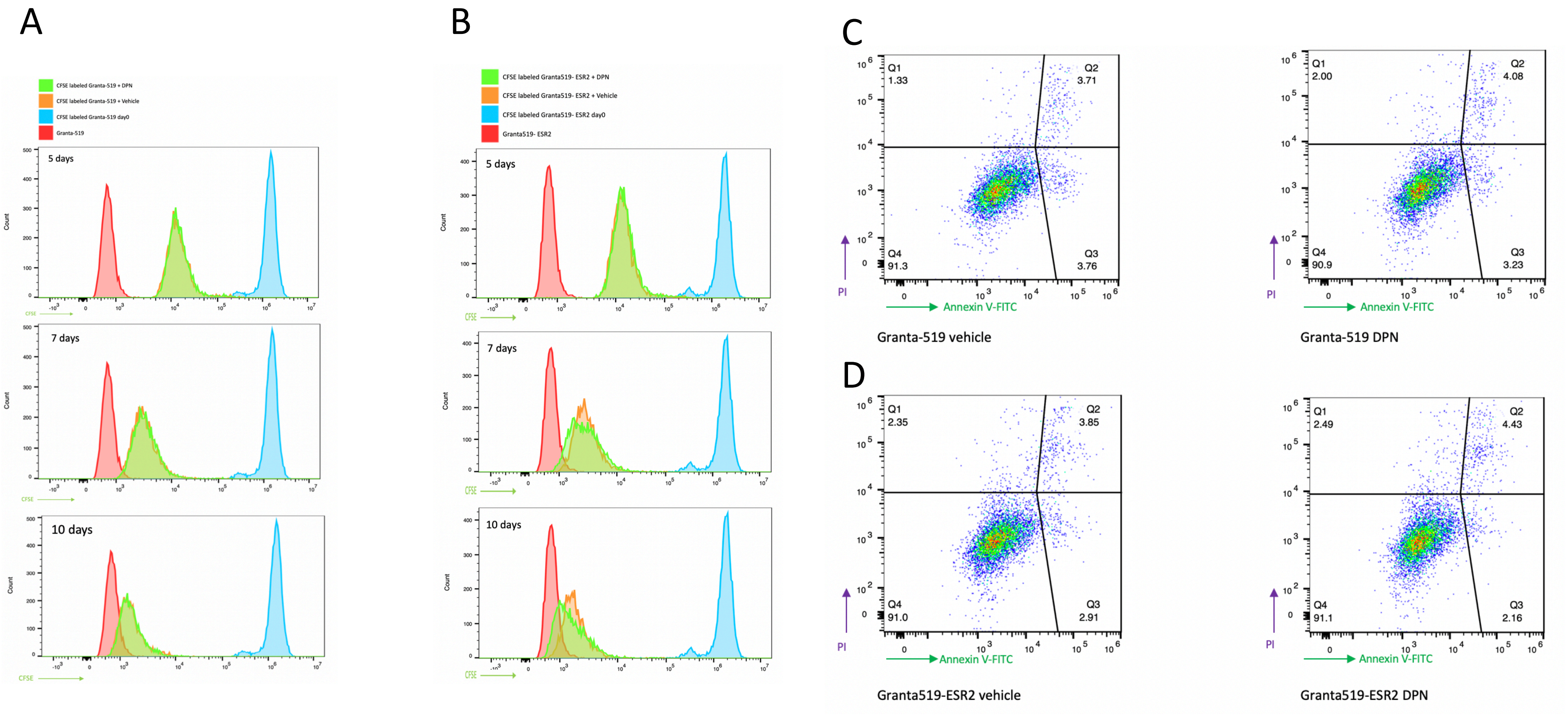

Supplementary figure S4: The effect of DPN treatment on proliferation and apoptosis of the Granta-519 and Granta519-ESR2 cells *in culture*. Granta-519 cells (S4A) and Granta519-ESR2 cells (S4B) were labeled with CFSE followed by treatment with 100 nM DPN or vehicle for 5, 7 or 10 days. After harvesting, the CFSE signal was determined by flow cytometry. Non-CSFE labeled and CFSE-labeled cells day 0 were used as negative and positive controls, respectively. Granta-519 cells (S4C) and Granta519-ESR2 cells (S4D) were treated with 100 nM DPN or vehicle for 24h. After treatment, cells were collected and stained with Annexin V-FITC and propidium iodide (PI), followed by analysis using flow cytometry. The percentage of viable (Q4), apoptotic (Q3), late apoptotic (Q2) and necrotic (Q1) cells are shown.

Suppl. Figure S5

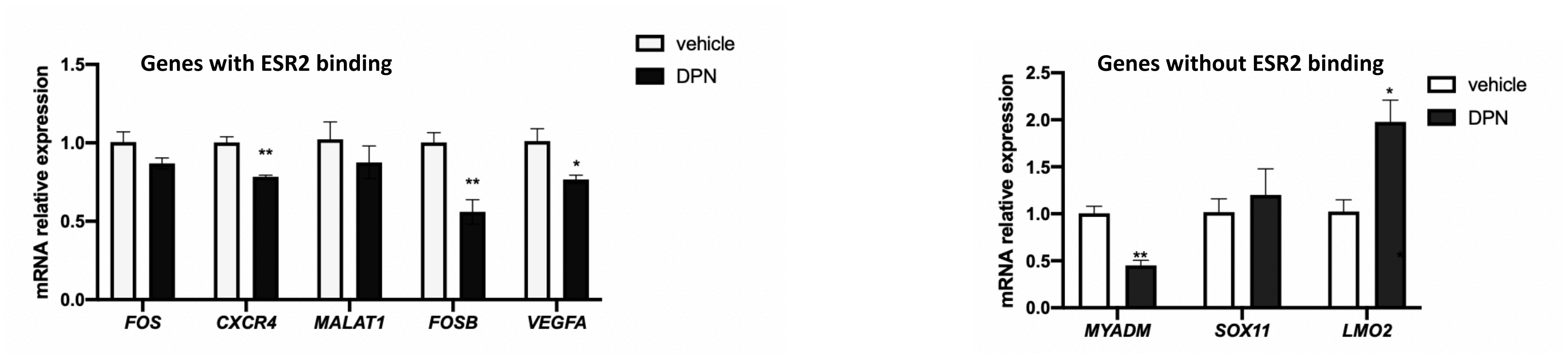

Supplementary figure S5: DPN regulates expression of a number of genes in Granta-519 cells in culture. Gene regulation of a selected number of genes in the wild-type Granta-519 cells following 24h treatment *in vitro* in the presence of 100 nM DPN or vehicle was analyzed by RT-qPCR. Data are represented as mean ± SD. Unpaired two-tailed t-test was used for statistical analysis between the two groups generated from separate experiments (n=3-5 per group, *p<0.05, **p<0.01).
