# Supplementary material for: Estrogen Receptor β (ESR2) Transcriptome and Chromatin Binding in a Mantle Cell Lymphoma Tumor Model Reveal the Tumor-Suppressing Mechanisms of Estrogens
Source: Cancers (Basel). 2022 Jun 24;14(13):3098. doi: 10.3390/cancers14133098 (PMC9264873; doi:10.3390/cancers14133098)
Supplement: Supplementary file 1 [file cancers-14-03098-s001.zip › Supplementary methods.pdf]

Supplementary methods

1. Dual luciferase reporter assay

2×10^6 Granta-519-ESR2 cells in 100 µl SF 4D-Nucleofector X solution (Lonza BioScience, Morrisville, NC, USA, Cat. No. V4XC-2012) were transfected with 2 µg 3 × ERE TATA luciferase plasmid (Addgene, Watertown, MA, USA, Cat. No. 11354) and 0.25 µg pRL-TK Renilla luciferase plasmid (Promega, Fitchburg, WI, USA, Cat. No. E2231) using program DN-113 on the 4D-Nucleofector X Unit (Lonza). 24 hours post transfection, cells were treated with 100 nM DPN or vehicle for 24h. Cells were then lysed and the luminescence of firefly and renilla luciferase activity were measured according to the instruction of Dual-luciferase reporter assay system (Promega, Cat. No. E1910).

2. Flow cytometry assay

For proliferation, cells were labeled with CFSE (Biolegend, San Diego, CA, USA, Cat. No. 423801) followed by treatment with 100 nM DPN or vehicle for 5 days, 7 days or 10 days respectively. Cell proliferation was measured by a reduced CFSE signal by flow cytometry. For analysis of apoptosis, cells were treated with 100 nM DPN or vehicle for 24h and stained with FITC-Annexin V and popidium iodide (PI) (Annexin V-FITC Apoptosis detection kit, Invitrogen, Waltham, Massachusetts, USA, Cat. no. BMS500FI-300). Analysis was performed by flow cytometry using a Beckman coulter Cytoflex S and the FACS data was analyzed by FlowJo.10.

3. Western blotting

Protein was extracted by using RIPA buffer (Sigma Aldrich, Burlington, MA, USA, Cat. No. R0278-50mL) with 1% protease inhibitor cocktail (PIC, Roche, Cat. No. 11873580001) according to the instruction. Western blotting was performed as previously described (1). Human ESR2 antibody PPZ0506 at 1:1000 dilution (R&D systems, Minneapolis, MN, USA, Cat. No. PP-PPZ0506-00) and monoclonal anti-β actin antibody (Sigma Aldrich, Cat. No. A5316-100ul) at 1:5000 dilution were used in this assay. The secondary antibody was horseradish peroxidase (HRP)-labeled anti-mouse IgG (GE Healthcare, Chicago, IL, USA, Cat. No. NA931) at 1:6000. BIO-RAD ChemiDoc Touch imaging system was used to quantify the signal.

4. Lentivirus transduction of Granta-519 cells

Before transduction, Granta-519 cells were seeded into a 24-well plate and maintained in phenol red free RPMI 1640 medium (GIBCO, Carlsbad, CA, USA, Cat. No. 11835-063) supplemented with 10% charcoal stripped fetal bovine serum (GIBCO, Cat. No. A33821-01). Granta-519 cells were stably transduced with a lentivirus containing an expression vector for the full length ESR2 (pLV[Exp]-Puro-EFIA>hESR2[NM_001437.2], 20 MOI. VectorBuilder Inc., Chicago, IL, USA) or a corresponding control lentivirus (pLV[Exp]-mPGK>puro, 2 MOI. VectorBuilder). Selection of stably transduced cells were obtained following treatment with 1.5 μg/ml puromycin (Sigma Aldrich, Cat. No. P8833-10mg) for 3 days in order to kill non-transduced cells. Stable Granta-519 cell lines with or without transduced full-length ESR2 expression (denoted Granta-519-ESR2 or Granta-519-mock), respectively, were cultured in phenol red free RPMI 1640 with 10% charcoal stripped FBS which contains low levels of steroids in order to exclude interference from culture medium in establishing stable cell lines.

5. Seq-ImmCC and CIBERSORTx

The transcriptional data of mouse genes was uploaded to Seq-ImmCC website (http://218.4.234.74:3200/immune/) and linear least square regression (LLSR) was selected to estimate the 10 different immune cell types. For CIBERSORTx (https://cibersortx.stanford.edu/), mouse gene symbols were converted to the corresponding human orthologue symbols since this program only processes HUGO nomenclature. LM22 (22 immune cell types) was chosen as the signature matrix file and the analysis was performed using 1000 permutations.

6. RNA-seq analysis

Bcl files were converted and demultiplexed to fastq using the bcl2fastq program. A hybrid human-mouse genome was created by merging the fasta files of the two organisms (hg38/mm10). Subsequently STAR (2) was used to index the hybrid reference genome and align the fastq files. Similarly, the exon annotations of the two organisms were merged and mapped reads were then counted using featureCounts (3). The entrez gene annotations and reference genome for both human and mouse were obtained from UCSC Genomic Institute. The count table from featureCounts was imported into R/Bioconductor and the gene counts originating from the two organisms were separated to their corresponding organism. Subsequent differential gene expression was performed separately on each organism using the EdgeR (4) package and its general linear models pipeline. For the gene expression analysis genes that had 1 count per million in 3 or more samples were used and normalized

using TMM (trimmed mean of M value) normalization. R package ClusterProfiler (5) was used for GSEA hallmark pathway analysis and GO biological processes pathway analysis.

7. ChIP-seq analysis

After using fastQC (6) to check the QC of ChIP-seq fastq.gz files, Trim-galore (7) was used to remove adapters and low-quality reads. Then ChIP-seq reads were aligned to UCSC human reference genome GRCh38 using Bowtie2 (8). The replicates were merged. Sequencing tags were read and imported using HOMER (9) followed by calling peaks with default settings and normalized to the input. Only peaks with FDR < 0.001 and fold enrichment over input tag count >4 (default settings) were kept. ChIPseeker (10) and HOMER were used for peaks annotation and drawing figures, and promoter regions defined as -3kb to 3kb. De novo motif analysis was performed by HOMER within +/- 100 bp from the peak center. HOMER was used to create bigwig files followed by uploading to UCSC Genomic Institute to show binding sites. Deeptools (11) was used to draw peaks' heatmaps.

8. TUNEL and Ki67 staining

Ki67 staining and TUNEL assay of tumor tissues were performed as described previously (12). ImageJ was used to count the number of stained cells from 10 randomly chosen fields (at 200× magnification) for each sample.

9. RNA extraction, cDNA synthesis and real-time quantitative PCR (RT-qPCR)

RNA was extracted with RNeasy Plus Mini Kit (Qiagen, Germantown, MD, USA, Cat No. 74136) according to the manufacture's instruction. cDNA synthesis and RT-qPCR were performed as described previously (13). Human RPLP0 (36B4) was used as the reference gene. The ΔΔCt method was used to calculate relative mRNA expression. Primer sequences used are shown in Supplementary Table S1.

10. ChIP-qPCR

Primers sequences for ChIP-qPCR are shown in Supplementary Table S1. ChIP-qPCR was performed on ABI Prism 7500 instrument using KAPA SYBR FAST (Sigma Aldrich, Cat. No. KK4605). Non-template negative control primers were used in all ChIP-qPCR as control. Each ChIP DNA sample was normalized against the

corresponding input sample and the percentage of input was used to report results. Unpaired two-tailed t-test was used for statistical analysis between samples treated with DPN and vehicle, respectively.

Reference:

1. Andersson S, Sundberg M, Pristovsek N, Ibrahim A, Jonsson P, Katona B, et al. Insufficient antibody validation challenges oestrogen receptor beta research. Nat Commun. 2017;8:15840.

2. Dobin A, Davis CA, Schlesinger F, Drenkow J, Zaleski C, Jha S, et al. STAR: ultrafast universal RNA-seq aligner. Bioinformatics. 2013;29(1):15-21.

3. Liao Y, Smyth GK, Shi W. featureCounts: an efficient general purpose program for assigning sequence reads to genomic features. Bioinformatics. 2014;30(7):923-30.

4. Robinson MD, McCarthy DJ, Smyth GK. edgeR: a Bioconductor package for differential expression analysis of digital gene expression data. Bioinformatics. 2010;26(1):139-40.

5. Yu G, Wang LG, Han Y, He QY. clusterProfiler: an R package for comparing biological themes among gene clusters. OMICS. 2012;16(5):284-7.

6. Andrews S. A quality control tool for high throughput sequence data 2019 [Available from: https://www.bioinformatics.babraham.ac.uk/projects/fastqc/.

7. Krueger F. A wrapper tool around Cutadapt and FastQC to consistently apply quality and adapter trimming to FastQ files, with some extra functionality for Mspl-digested RRBS-type (Reduced Representation Bisufite-Seq) libraries. 2019 [Available from: https://www.bioinformatics.babraham.ac.uk/projects/trim_galore/.

8. Langmead B, Salzberg SL. Fast gapped-read alignment with Bowtie 2. Nat Methods. 2012;9(4):357-9.

9. Heinz S, Benner C, Spann N, Bertolino E, Lin YC, Laslo P, et al. Simple combinations of lineage-determining transcription factors prime cis-regulatory elements required for macrophage and B cell identities. Mol Cell. 2010;38(4):576-89.

10. Yu G, Wang LG, He QY. ChIPseeker: an R/Bioconductor package for ChIP peak annotation, comparison and visualization. Bioinformatics. 2015;31(14):2382-3.

11. Ramirez F, Ryan DP, Gruning B, Bhardwaj V, Kilpert F, Richter AS, et al. deepTools2: a next generation web server for deep-sequencing data analysis. Nucleic Acids Res. 2016;44(W1):W160-5.

12. Talaber G, Yakimchuk K, Guan J, Inzunza J, Okret S. Inhibition of estrogen biosynthesis enhances lymphoma growth in mice. Oncotarget. 2016;7(15):20718-27.

13. Qiao S, Chen L, Okret S, Jondal M. Age-related synthesis of glucocorticoids in thymocytes. Exp Cell Res. 2008;314(16):3027-35.
