# Supplementary material for: Estrogen Receptor β (ESR2) Transcriptome and Chromatin Binding in a Mantle Cell Lymphoma Tumor Model Reveal the Tumor-Suppressing Mechanisms of Estrogens
Source: Cancers (Basel). 2022 Jun 24;14(13):3098. doi: 10.3390/cancers14133098 (PMC9264873; doi:10.3390/cancers14133098)
Supplement: Supplementary file 1 [file cancers-14-03098-s001.zip › Supplementary Table S1.pdf]

Supplementary table 1

Primers for RT-qPCR

	**Forward Sequence (5'-3')**	**Reverse Sequence (5'-3')**
MALAT1	AAAGCAAGGTCTCCCCACAAG	GGTCTGTGCTAGATCAAAAGGCA
VEGFA	GGGCCTCCGAAACCATGAA	AGCTGCGCTGATAGACATCC
SOX11	GACGGTCAAGTGCGTGTTTC	GCACTTTGGCGACGTTGTAG
FOSB	CTGACCGACCGACTCCAG	AAATCTCTCACCTCCGCCAG
MYADM	AGCGCGGTGCTCTTACAG	GAAGATGACGTCGTGGTGGT
CXCR4	CGTCTCAGTGCCCTTTTGTTC	CTGAAGTAGTGGGCTAAGGGC
JUN	GCCAGGTCGGCAGTATAGTC	TCTGGACACTCCCGAAACAC
FOS	TACTACCACTCACCCGCAGA	CGTGGGAATGAAGTTGGCAC
NEAT1	GGAGAGGGTTGGTTAGAGAT	CCTTCAACCTGCATTTCCTA
LMO2	AAGCAGGCAATTAGCCCAGA	GCTCAGCTTCCCTCTGTCTC
RPLP0	TTCTCGCTTCCTGGAGGGTG	GACAAGGCCAGGACTCGTTT

Primers for ChIP-qPCR

	**Forward Sequence (5'-3')**	**Reverse Sequence (5'-3')**
GREB1	AGCAGTGAAAAAAAGTGTGGCAACTGGG	CGACCCACAGAAATGAAAAGGCAGCAAACT
VEGFA	AGGGGCTTTCAAGGTAACTCC	CCACCAAGGCTACAGAGAGAC
CXCR4	CTATCCCCGGAGCGCAAAT	TTCGAGAGTTTGGGGTCGTG
MALAT1	TCACGGCCCTCGCGTAT	TCCTTTACAGAAGTCTCGGGCT
NEAT1	CAGGGTGCTCAGTGACCTTT	GGTTTCGGTCTGTTTTGCCC
FOS	GAAATCCCTGCCCTGTTGGAA	AGGGTGACTTTGAGACAGGTG
FOSB	CAGAGGTTGCAGTGACACAA	GCTAACCTTCTGACCCCTTACT
Negative control	GGGGGATCAGATGACAGTAAA	AATGCCAGCATGGGAAATA
